# An Optimized Vector Tracking Architecture for Pseudo-Random Pulsing CDMA Signals

**DOI:** 10.3390/s21124087

**Published:** 2021-06-14

**Authors:** Lin Tao, Guangchen Li, Junren Sun, Bocheng Zhu

**Affiliations:** School of Electronics Engineering and Computer Science, Peking University, Beijing 100871, China; taolin_yiran@pku.edu.cn (L.T.); liguangchen@pku.edu.cn (G.L.); sunjunren@pku.edu.cn (J.S.)

**Keywords:** pseudo-random pulsing signal, irregular update periods, predicted mesurement, vector tracking loop

## Abstract

The vector tracking loop (VTL) has high tracking accuracy and a superior ability to track weak signals in GNSS. However, traditional VTL architecture is established on continuous Code Division Multiple Access (CDMA) signal and is incompatible with pseudolite positioning systems (PLPS) because PLPS generally adopts a pseudo-random pulsing CDMA signal structure to mitigate the near-far effect. Therefore, this paper proposes an optimized VTL architecture for pseudo-random pulsing CDMA signals. To avoid estimation biases in PLPS, the proposed VTL adopts irregular update periods (IUP) pre-filters which adjust the update cycles according to the active timeslot intervals. Meanwhile, as the active timeslots of different pseudolites do not overlap, the sampling time of the navigation filter inputs is inconsistent and time-varying, causing jitter degradation. Thus, the proposed VTL predicts the measurements so that they can be sampled at the same time, which improves tracking accuracy. Simulation is carried out to evaluate the performance of the proposed VTL. The results suggest that the proposed VTL outperforms the traditional pre-filter-based VTL and IUP pre-filter-based VTL.

## 1. Introduction

The Global Navigation Satellite System (GNSS) has been considered the first alternative to provide real-time positioning, navigation, and timing (PNT) services worldwide. Despite the benefit of broad coverage, high accuracy, and low cost, the GNSS-based PNT systems are fragile in challenging scenarios, such as deep valleys, heavily forested areas, open-cut mines, urban canyons, and even indoors [1,2]. Therefore, much research has been carried out to improve GNSS availability and robustness in these challenging environments [3,4]. The studies can be separated into two directions. One is to improve the performance of GNSS systems [3], such as adopting more advanced signal structures [5,6], applying anti-jamming receiving technologies [7], and enhancing signal power. The other is to design new positioning systems suitable for the ground [4,8], such as ultra-wideband [9], Wi-FI [10], 4G or 5G [11], and pseudolite-based positioning technologies [12,13,14]. Due to the benefits of the great flexibility, easy servicing, broad signals coverage, compatibility with GNSS, and high positioning precision, the pseudolite positioning system has attracted increasing attention in recent years.

Pseudolites are ground-based satellites transmitting GNSS-like signals. The positioning precision and continuity of the GNSS can effectively be improved by installing pseudolites in urban areas, indoors, and in scenarios where the GNSS is problematic. Simultaneously, pseudolites can also be used to offer continuous and reliable positioning services to users as an independent positioning system. However, the Pseudolites Positioning Systems (PLPS) suffers severely from the near-far effect [15,16]. The near-field pseudolites may interfere with far-field signals when the receiver moves close to a pseudolite, causing failure to track and synchronize the distant pseudolites signal, referred to as the near-far effect [16]. Various techniques have been proposed for alleviating the near-far effect, including frequency hopping [17], new spreading codes [15] pulse modulation [18], and optimizing pseudolite antenna [19]. However, the preferred one is to pulse the pseudolite signals in pseudo-random because of the low resource cost and good resistance to the near-far effect.

As the name indicates, the pulse signals could be regarded as gated direct sequence spread spectrum (DSSS) signals. Each pseudolite transmits signals at different timeslots, preventing mutual interference. Although the pulse signal with equal intervals is easier to realize, it may result in sub-peaks in the frequency domain, causing false frequency-lock in the receiver [20,21]. Thus, the pseudo-random pulse scheme should be adopted in PLPS. Some literature [18,22,23] offer different designs of the pulse scheme. The cornerstone of these schemes is to use a pseudo-random pulse sequence to control pseudolite pulse signal transmission.

The pseudo-random pulse signal (PRPS) brings some challenges in tracking compared to GNSS signals. It would increase the measurement noise and decrease the tracking convergence speed if the receiver tracks the PRPS according to the traditional tracking architecture. A tracking loop architecture of conventional PLL and delay lock loop (DLL) pre-cascaded with a Noise Silencer Module (NSM) is proposed in Reference [24]. This tracking loop is turned on only when the valid timeslot of the signal arrives. It mitigates the interference of other pseudolite signals and reduces noise accumulation when the signal is silent. Because the intervals between two adjacent valid timeslots are pseudo-random, the discriminator of the NSM-PLL/DLL tracking loop has a bias in estimating the carrier phase and code phase, reducing the tracking accuracy of the loop. Therefore, Yun et al. propose a variable update rate carrier tracking loop based on the Kalman filter (KF-VURL) [25], which effectively solves the estimation bias problem of the discriminator. In estimating the carrier phase and doppler, the KF-VURL shows higher tracking accuracy and faster convergence.

Though much research on pseudolite tracking focuses on improving and optimizing the scalar tracking loop (STL) [24,25,26,27], they ignore the abundant information of other channels. In contrast, the VTL uses a big loop to filter the observations of all channels, which makes full use of the information of each channel and improves the tracking performance of the signals. Initially, the VTL was introduced by Spilker [28] and popularly developed in recent years, mainly applied in GNSS. The VTL algorithm can track weak signals, which increases the number of visible satellites and, thus, has better performance in harsh environments [29]. Furthermore, the VTL algorithm utilizes other normal operation tracking channels to predict and compensate for the observations of momentary outage signals to maintain continuous tracking [30].

The typical types of VTL include vector delay lock loop (VDLL) [30,31,32], vector frequency lock loop (VFLL) [31,32], vector delay frequency lock loop (VDFLL) [32,33,34], and vector phase lock loop (VPLL) [35]. Hyoungmin So et al. propose a VTL algorithm applicable to asynchronous PLPS, significantly mitigating the near-far problem [36]. However, its VTL is still based on the continuous GNSS signal model, ignoring the pseudo-random pulsing characteristic of pseudolite signals. The measurement inputs of the navigation filter in traditional VTL are discriminator outputs in each channel, and the navigation filter obtains the errors at similar sampling time. Nevertheless, this structure is not appropriate for the vector tracking of pseudolite signals. The reasons are as follows. Firstly, the output of the discriminator can only reflect the errors before the valid timeslot due to the pulse feature of PRPS. Secondly, as the active timeslots of different pseudolites do not overlap, the output time of each channel discriminator is different.

The structure based on the pre-filters and navigation filter provides a possible solution to these problems in tracking pseudolite signals because, by improving the architecture of pre-filters, the robustness of VTL would be enhanced, and its application scope could be expanded. Unfortunately, the application of pre-filter-based VTL mainly focuses on GNSS signals. Jin et al. present a federal tracking loop consist of subfilters in every channel and a master filter [37]. By adding subfilters, the computational complexity of VTL is effectively reduced, and the robustness is also enhanced, while the tracking performance is almost unchanged. The pre-filters in the VTL proposed by Xie et al. are based on the unscented Kalman filter (UKF) and directly operate on the in-phase and quadra-phase correlator outputs to obtain the phase and frequency biases [38]. However, these pre-filters are based on the continuous GNSS model, causing jitter degradation when tracking PRPS.

To solve the problems in tracking PRPS, an optimized VTL architecture that includes irregular update periods (IUP) pre-filters and a navigation filter based on predicted measurements (PM) is proposed. The proposed VTL is abbreviated as IUPPM-VTL. According to the timeslot intervals, the IUP pre-filter based on the Kalman filter (KF) adjusts the update cycles, avoiding the impact of the pseudo-random pulsing signal. So, it can estimate the measurement accurately. However, since the pre-filters are updated at varying timeslots and not consistent with each other, using them directly as the inputs of the navigation filter would increase the vector tracking estimation noise. As a consequence, the IUPPM-VTL predicts the measurements so that they can be to sampled at the same time. Meanwhile, this paper compares the proposed IUPPM-VTL with traditional VTL based on pre-filter and IUP-VTL based on IUP-prefilter.

The rest of this paper is organized as follows. Section 2 introduces the PRPS structure. Section 3 describes the problems in tracking PRPS. Section 4 proposes the optimized VTL architecture. The simulation and comparison testing are carried out in Section 5. Conclusions and future work are summarized in Section 6.

## 2. Pseudo-Random Pusling Signal Structure

**Figure 1:**
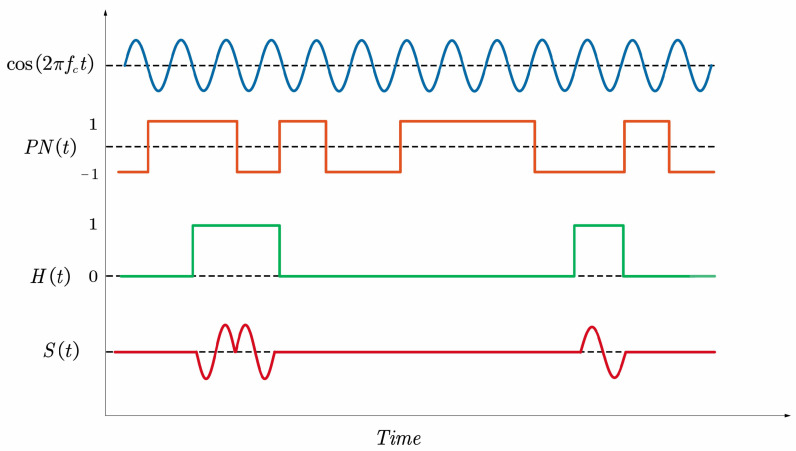
Schematic diagram of PRPS modulation.

The signal structure of PRPS is first introduced. The pseudolite positioning system is a ground-based satellite navigation system that uses a low-cost launcher to transmit GNSS-like signals. Therefore, the pseudolite system relies on the spreading code to achieve terminal equipment positioning functions. The difference is that the pseudolite signal adopts the pulsing signal structure to overcome the near-far effect. Figure 1 shows the modulation process of PRPS, which is equivalent to the pulse modulation of a continuous CDMA signal.

The mathematical model of the received pseudo-random pulsing signal is expressed by the following formula.

(1) $$S^{i}\left(t\right)=\left[A^{i}D^{i}\left(t\right)PN^{i}\left(t-\tau^{i}\right)\cos\left(2\pi \left(f_{IF}+f_{d}^{i}\right)t+\varphi_{0}^{i}\right)\right]\cdot H^{i}\left(t-\tau^{i}\right),$$

where $H^{i}\left(t\right)$ is the pulse modulation sequences. The pseudo-random code for the *i*-th pseudolite $A^{i}$ represents amplitude of the received signal, $D^{i}\left(t\right)$ denotes the modulated data bits, $\tau^{i}$ is the *i*-th transmitter-receiver time delay, $f_{d}^{i}$ is the doppler shift, and $f_{IF}$ and $\varphi_{0}^{i}$ are the intermediate frequecy (IF) and initial carrier phase, respectively.

The pulse signal $H^{i}\left(t\right)$ at the *i*-th transmitter is repeated by the basic pulse scheme $h_{0}^{i}\left(t\right)$, which can be modeled as follows:

(2) $$H^{i}\left(t\right)=\sum_{m=0}^{+\infty }h_{0}^{i}\left(t-mT_{p}\right),$$

where $T_{p}$ represents the repetition period of $h_{0}^{i}\left(t\right)$, which can be expressed as:

(3) $$h_{0}^{i}\left(t\right)=\sum_{k=0}^{N_{f}-1}\Omega \left(t-kT_{f}-c_{k}^{i}T_{s}\right).$$

Ω(t) is the single square wave function, which is

(4) $$\Omega \left(t\right)=\left\{\begin{array}{cc} 1, & t\in \left[0,T_{s}\right)\\ 0, & \;\mathrm{otherwise}\; \end{array}.\right.$$

In Equation (3), $T_{s}$ denotes the duration of timeslot, $T_{f}$ is one frame time which is equal to $N_{s}T_{s}$, and $N_{s}$ is the number of timeslots in one frame. The pulse duty cycle *d* is $1/N_{s}$, and $N_{f}$ indicates the frame number of the basic pulse pattern, so $T_{p}=N_{f}T_{f}$. $c_{k}^{i}$ represents the pulse position index in *k*-th frame. Meanwhile, $c_{k}^{i}$ satisfies the following two requirements:

1. The value of $c_{k}^{i}$ ranges from 0 to $N_{s}-1$.
2. The active timeslots of different pseudolites do not overlap.

Generally, $c_{k}^{i}$ is a pseudo-random permutation. It ensures the pulse pseudolite signal has better spectral properties, described in detail in Reference [18]. The pseudolite transmits signals in active timeslots where $H^{i}\left(t\right)$ is 1 and keeps silent in silent timeslots where $H^{i}\left(t\right)$ is 0. Thus, the pseudolite signals would not interfere with each other. The transmitting time of the pulse signal is time-varying and predetermined by a pseudo-random permutation.

**Figure 2:**
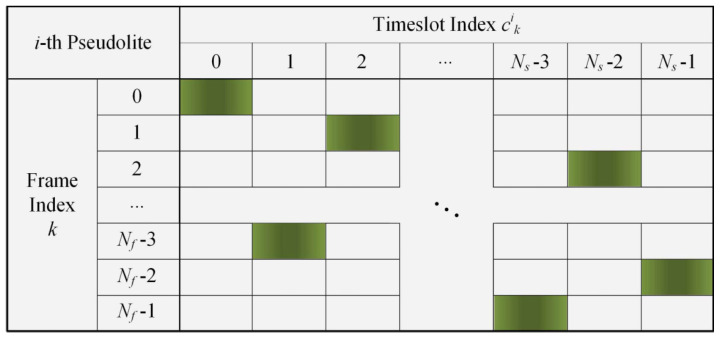
Illustration of the pulse pattern.

An illustration of the pulse pattern is shown in Figure 2. The horizontal axis represents timeslot index $c_{k}^{i}$ ranging from 0 to $N_{s}-1$ in one frame, and the vertical axis represents the frame index *k* ranging from 0 to $N_{f}-1$. The green squares represent the active timeslots indexes.

## 3. Problem Description

Based on the PRPS structure, this section analyzes the problems existing in the vector tracking process of pseudolite signals. The local replica $S_{r}^{i}\left(t\right)$ of *i*-th pseudolite is defined as:

(5) $$S_{r}^{i}\left(t\right)=PN_{r}^{i}\left(t\right)\cos\left(2\pi f_{r}t\right)\cdot H_{r}^{i}\left(t\right),$$

where $PN_{r}^{i}$ represents the spreading code replica, $H_{r}^{i}$ denotes the pseudo-random pulsing signal replica, and $f_{r}$ is the frequency of local replica.

The in-phase (I) integration of prompt correlator can be described in the following formula when the receiver tracks the signal of *i*-th pseudolite.

(6) $$\begin{array}{cc} I_{p}\left(k\right) & =\frac{1}{T_{s}}\int_{t_{k}}^{t_{k}+T_{s}}S^{i}\left(t\right)\cdot S_{r}^{i}\left(t\right)\;dt\\ \; & =AD\left(k\right)R\left(\Delta \tau_{k}\right)\mathrm{sinc}\left(\Delta f_{k}T_{s}\right)\cos\left[2\pi \Delta f_{k}\left(t_{k}+\frac{1}{2}T_{s}\right)+\Delta \phi_{0}\right]+n_{I} \end{array},$$

where $\Delta \tau_{k}$ and $\Delta f_{k}$ denote the code phase and the frequency errors between the received signal and the local replica in the current epoch, respectively. $\Delta \phi_{0}$ is the initial carrier phase error. R(·) is the autocorrelation value of the spreading code, $T_{s}$ is timeslot period, and $n_{I}$ indicates the in-phase noise. The starting integration time $t_{k}$ is

(7) $$t_{k}=kT_{f}+c_{k}^{i}T_{s}=\left(kN_{s}+c_{k}^{i}\right)\cdot T_{s}.$$

The prompt quadra-phase (Q) integration of prompt correlator can be expressed as:

(8) $$Q_{p}\left(k\right)=AD\left(k\right)R\left(\Delta \tau_{k}\right)\mathrm{sinc}\left(\pi \Delta f_{k}T_{s}\right)\sin\left[2\pi \Delta f_{k}\left(t_{k}+\frac{1}{2}T_{s}\right)+\Delta \phi_{0}\right]+n_{Q}.$$

The average carrier phase error measured by the discriminator is as follows.

(9) $$\bar{\Delta \phi_{k}}=\arctan\left(\frac{Q_{p}}{I_{p}}\right)≃2\pi \Delta f_{k}\left(t_{k}+\frac{1}{2}T_{s}\right)+\Delta \phi_{0}.$$

The discriminator output approximates the average phase error between the received signal and the local replica in the active timeslot, as defined in Equation (9). Thus, $\bar{\Delta \phi_{k}}$ is affected by $\Delta f_{k}$, $T_{s}$, $\Delta \phi_{0}$, and $t_{k}$. Since the tracking process replicates the signal parameters by filtering the discriminator outputs, it is vital to establish an appropriate output model. For simple analysis, this paper supposes that the tracking loop does not feedback the estimations to NCO. Thus, $\Delta f_{k}$ would be constant in this process. $\Delta \phi_{0}$ is the initial carrier phase error, and $T_{s}$ is the timeslot period. Both of them are constant, while $t_{k}$ is varying. Hence, it is necessary to focus on the analysis of the influence of $t_{k}$ on $\bar{\Delta \phi_{k}}$. Within each tracking period, the variation of carrier phase discriminator output can be expressed as:

(10) $$\nabla \bar{\Delta \phi_{k}}=\bar{\Delta \phi_{k}}-\bar{\Delta \phi_{k-1}}=2\pi \Delta f_{k}T_{k-1},$$

where

(11) $$T_{k-1}=t_{k}-t_{k-1}=\left(N_{s}+c_{k}^{i}-c_{k-1}^{i}\right)\cdot T_{s}.$$

**Figure 3:**
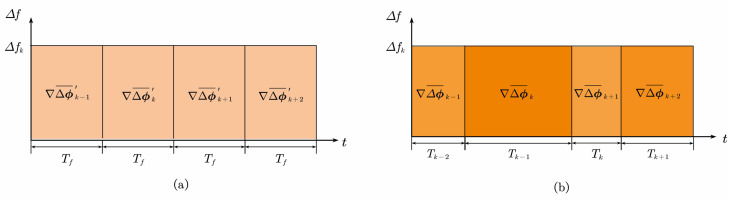
The comparision between $\nabla \bar{\Delta \phi_{k}}$ in tracking continuous CDMA signal and PRPS. (**a**) $\nabla \bar{\Delta \phi \prime_{k}}$ of tracking continuous CDMA signal; (**b**) $\nabla \bar{\Delta \phi_{k}}$ of tracking PRPS.

$T_{k-1}$ represents the intervals between the last active timeslot and the currently active timeslot, which is time-varying and predetermined by the pseudo-random permutation. However, for continuous CDMA signal, $T_{k-1}$ is equal to the constant correlation integration time $T_{f}$. The difference of $\nabla \bar{\Delta \phi_{k}}$ in tracking continuous CDMA signal and PRPS is shown in Figure 3.

The horizontal axis in Figure 3 represents the time, and the vertical axis represents the frequency error, so the shaded area represents $\nabla \bar{\Delta \phi_{k}}$ (ignoring the scaling factor 2π). The $\nabla \bar{\Delta {\phi^{\prime }}_{k}}$ is time-invariant in tracking continuous CDMA signal shown in Figure 3a. Thus, with a fixed update period, the traditional tracking loop could accurately estimate and correct the frequency error, whereas Figure 3b shows that, when tracking PRPS, the $\nabla \bar{\Delta \phi_{k}}$ is time-variant because of $T_{k-1}$. It is supposed that the time-varying property of $T_{k}$ is ignored in the tracking loop design. In that case, the frequency error cannot be accurately estimated, resulting in jitter degradation of tracking performance, which is also described in Reference [25].

Similarly, the code phase discriminator output is also time-varying as the code phase error is driven by the same, albeit scaled, dynamics as the carrier phase over short time intervals [39]. The variation of code phase discriminator output is

(12) $$\begin{array}{cc} \nabla \bar{\Delta \tau_{k}} & =\beta \Delta f_{k}T_{k-1}\\ \beta & =\frac{f_{code}}{f_{c}} \end{array},$$

where $f_{code}$ indicates the frequency of spreading code.

According to Equations (9), (10), and (12), the error parameter is related to the time intervals $T_{k-1}$ of adjacent effective timeslots. These parameters can be accurately estimated by designing a scalar tracking loop of variable update period, as researched in References [24,25,27].

**Figure 4:**
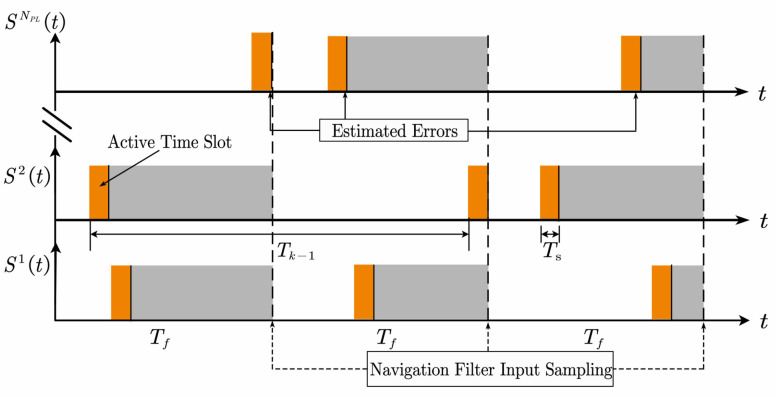
Schematic diagram of input sampling for navigation filter.

In the vector tracking structure, the input observations of the navigation filter need to be sampled simultaneously. However, the estimation time of the errors in each channel is inconsistent and time-varying in PLPS. Figure 4 depicts this problem. The dotted arrows represent the navigation filter sampling time, while the solid arrows indicate the error estimation time in each channel. Unfortunately, there exists a time difference between them. Therefore, the input observations of the navigation filter cannot reflect the errors during this time (gray shaded part). Even worse, this time difference is pseudo-random, causing jitter degradation of the navigation filter when using conventional VTL loops to track PRPS. Hence, it is necessary to design the VTL according to the characteristics of the PRPS.

## 4. The Proposed VTL Architecture

Based on the analysis of Section 3, it can be concluded that, if the VTL is used to track the PRPS with the architecture of a continuous CDMA signal, the performance of the VTL will degrade due to the signal structure difference. To solve the problems in tracking PRPS, an optimized VTL architecture that includes irregular update periods (IUP) pre-filters and a navigation filter based on predicted measurements (PM) is proposed. This section first presents the architecture of the IUPPM-VTL and then discusses the discrete-time models of the pre-filter and the navigation filter.

### 4.1. Architecture of the IUPPM-VTL

**Figure 5:**
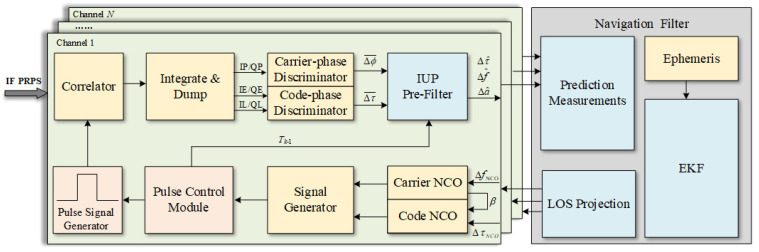
Block diagram of the IUPPM-VTL.

Figure 5 shows the block diagram of the IUPPM-VTL. The pulse control module generates the pulse signal $H^{i}\left(t\right)$ and calculates intervals $T_{k-1}$ between two adjacent active timeslots according to the predetermined pseudo-random permutation. The Integrate and Dump (I&D) module only integrates during the active timeslots, avoiding interference of other PRPS. The discriminator outputs the carrier phase error $\bar{\Delta \phi_{k}}$ and the code phase error $\bar{\Delta \tau_{k}}$. Then, the IUP pre-filter based KF filters $\bar{\Delta \phi_{k}}$ and $\bar{\Delta \tau_{k}}$ in irregular periods according to the intervals $T_{k-1}$. Meanwhile, the estimated errors of the pre-filter are the inputs of the navigation filter. The input measurements are firstly predicted to ensure the same sampling time. Thus, it will not lead to performance degradation due to inconsistent input sampling times. Finally, the corrections estimated by EKF are used to adjust the channel NCO through the line of sight (LOS) projection.

### 4.2. Pre-Filter Model

Because the update period of the states in KF can be time-varying, while that in PLL and DLL can only be fixed, the KF structure is adopted for the IUP pre-filter.

The state vector of linear KF describes the system time evolution [40] and is typically defined as an error vector of four parameters: code phase error Δτ (unit: chips), carrier phase Δφ (unit: radians), carrier frequency error Δf (unit: Hz), and carrier frequency rate error Δa (unit: Hz/s). The state vector at the *k*-th active timeslot can be described as:

(13) $$\Delta X_{k,1}={\left[\Delta \tau ,\Delta \varphi ,\Delta f,\Delta a\right]}_{k}^{T},$$

where subscript 1 indicates the pre-filter.

The dynamic system model is related to the intervals, described in Equation (11).

(14) $$\Delta X_{k,1}=F_{k,1}\cdot \Delta X_{k-1,1}+w_{k-1,1},$$

where $F_{k,1}$ denotes the state transition matrix [39,41] as:

(15) $$F_{k,1}={\left[\begin{array}{cccc} 1 & 0 & \beta T_{k-1} & \frac{1}{2}\beta T_{k-1}^{2}\\ 0 & 1 & 2\pi T_{k-1} & \pi T_{k-1}^{2}\\ 0 & 0 & 1 & T_{k-1}\\ 0 & 0 & 0 & 1 \end{array}\right]}_{k}.$$

It should be emphasized that $T_{k-1}$ is not constant. Instead, it is calculated according to the pseudo-random active timeslot intervals, avoiding the inaccurate output model of the discriminators, as described in Section 3.

In Equation (14), $w_{k-1,1}$ indicates the process noise vector, and its covariance matrix is $Q_{k-1,1}$ which can be described as [39,40]:

(16) $$Q_{k-1,1}=Q_{code}+Q_{LOS}+Q_{p}+Q_{f},$$

where

(17) $$Q_{code}=q_{code}\left[\begin{array}{cccc} T_{k-1} & 0 & 0 & 0\\ 0 & 0 & 0 & 0\\ 0 & 0 & 0 & 0\\ 0 & 0 & 0 & 0 \end{array}\right],\;Q_{\mathrm{LOS}}=q_{\mathrm{LOS}}\left[\begin{array}{cccc} \frac{T_{k-1}^{5}}{20}\beta^{2} & \frac{T_{k-1}^{5}}{20}\beta^{2} & \frac{T_{k-1}^{4}}{8}\beta & \frac{T_{k-1}^{3}}{6}\beta \\ \frac{T_{k-1}^{5}}{20}\beta^{2} & \frac{T_{k-1}^{5}}{20} & \frac{T_{k-1}^{4}}{8} & \frac{T_{k-1}^{3}}{6}\\ \frac{T_{k-1}^{4}}{8}\beta & \frac{T_{k-1}^{4}}{8} & \frac{T_{k-1}^{3}}{3} & \frac{T_{k-1}^{2}}{2}\\ \frac{T_{k-1}^{3}}{6}\beta & \frac{T_{k-1}^{3}}{6} & \frac{T_{k-1}^{2}}{2} & T_{k-1} \end{array}\right],$$

(18) $$Q_{p}=S_{p}\omega_{RF}^{2}\cdot \left[\begin{array}{cccc} T_{k-1}\beta^{2} & T_{k-1}\beta & 0 & 0\\ T_{k-1}\beta & T_{k-1} & 0 & 0\\ 0 & 0 & 0 & 0\\ 0 & 0 & 0 & 0 \end{array}\right],\;Q_{f}=S_{f}\omega_{RF}^{2}\cdot \left[\begin{array}{cccc} \frac{T_{k-1}^{3}}{3}\beta^{2} & \frac{T_{k-1}^{3}}{3}\beta & \frac{T_{k-1}^{2}}{2}\beta & 0\\ \frac{T_{k-1}^{3}}{3}\beta & \frac{T_{k-1}^{3}}{3} & \frac{T_{k-1}^{2}}{2} & 0\\ \frac{T_{k-1}^{2}}{2}\beta & \frac{T_{k-1}^{2}}{2} & T_{k-1} & 0\\ 0 & 0 & 0 & 0 \end{array}\right],$$

where $q_{LOS}$ represents the random walk process driving the line-of-sight acceleration, $q_{code}$ represents the code/carrier divergence, and $S_{p}$ and $S_{f}$ denote the phase and frequency random walk power spectral density due to the receiver oscillator, respectively. $w_{RF}$ is the carrier frequency. Given the oscillator h-parameters, the clock noise spectral densities are obtained as [39,42]:

(19) $$\begin{array}{cc} S_{p} & =\frac{1}{2}h_{0}\\ S_{f} & =2\pi^{2}h_{-2} \end{array}.$$

The discriminator outputs of the code phase $\bar{\Delta \tau }$ and the carrier phase $\bar{\Delta \phi }$ are used as the measurements in the pre-filter. So, the measurement vector $Z_{k,1}$ is:

(20) $$Z_{k,1}={\left[\;\bar{\Delta \tau },\;\bar{\Delta \phi }\;\right]}_{k}^{T}.$$

The measurement model can be expressed as follows:

(21) $$Z_{k,1}=H_{1}\cdot \Delta X_{k,1}+v_{k,1},$$

where $v_{k}$ is the measurement noise, and its covariance matrix $R_{k,1}$ is

(22) $$R_{k,1}=\left[\begin{array}{cc} \sigma_{\bar{\Delta \tau }}^{2} & 0\\ 0 & \sigma_{\bar{\Delta \phi }}^{2} \end{array}\right],$$

where $\sigma_{\bar{\Delta \tau }}^{2}$ and $\sigma_{\bar{\Delta \phi }}^{2}$ are the noise standard deviations of the code phase and carrier phase discriminator outputs, respectively. The early minus late envelope and the four-quadrant arctangent are selected as code and carrier discriminators. The measurement variances for the above discriminators are given by References [39,41]:

(23) $$\sigma_{\Delta \tau }^{2}=\frac{d}{4C/N_{0}\cdot T_{s}}\cdot \left[1+\frac{2}{\left(2-d\right)C/N_{0}\cdot T_{s}}\right],$$

(24) $$\sigma_{\Delta \phi }^{2}=\frac{1}{2C/N_{0}\cdot T_{s}}\cdot \left(1+\frac{1}{2C/N_{0}\cdot T_{s}}\right),$$

where *d* is the spacing between early and late replica codes, and $C/N_{0}$ is the carrier-to-noise ratio.

The relationship between the states and the measurements can be modeled as follows:

(25) $$\begin{array}{cc} \bar{\Delta \tau_{k}} & =\frac{1}{T_{s}}\int_{T_{k-1}}^{T_{k-1}+Ts}\left(\Delta \tau_{k-1}+\beta \Delta f_{k-1}t+\frac{1}{2}\beta \Delta a_{k-1}t^{2}\right)dt\\ \; & =\Delta \tau_{k}-\frac{1}{2}\beta \Delta f_{k}T_{s}+\frac{1}{6}\beta \Delta a_{k}T_{s}^{2} \end{array},$$

(26) $$\begin{array}{cc} \bar{\Delta \phi_{k}} & =\frac{1}{T_{s}}\int_{T_{k-1}}^{T_{k-1}+Ts}\left(\Delta \varphi_{k-1}+2\pi \Delta f_{k-1}t+\pi \Delta a_{k-1}t^{2}\right)dt\\ \; & =\Delta \varphi_{k}-\pi \Delta f_{k}T_{s}+\frac{1}{3}\pi \Delta a_{k}T_{s}^{2} \end{array}.$$

According to Equations (13), (25), and (26), the measurement model can be reconstructed as:

(27) $${\left[\begin{array}{c} \bar{\Delta \tau_{k}}\\ \bar{\Delta \phi_{k}} \end{array}\right]}_{k}=\left[\begin{array}{cccc} 1 & 0 & -\frac{1}{2}\beta T_{s} & \frac{1}{6}\beta T_{s}^{2}\\ 0 & 1 & -\pi T_{s} & \frac{1}{3}\pi T_{s}^{2} \end{array}\right]\cdot {\left[\begin{array}{c} \Delta \tau \\ \Delta \varphi \\ \Delta f\\ \Delta a \end{array}\right]}_{k}+v_{k,1}.$$

Thus, the observation matrix $H_{1}$ in Equation (21) is

(28) $$H_{1}=\left[\begin{array}{cccc} 1 & 0 & -\frac{1}{2}\beta T_{s} & \frac{1}{6}\beta T_{s}^{2}\\ 0 & 1 & -\pi T_{s} & \frac{1}{3}\pi T_{s}^{2} \end{array}\right].$$

After establishing the dynamic and measurement models, the KF including one-step prediction and measurement correction can be implemented [40].

- Prediction
  (29) $$\begin{array}{cc} \Delta {\hat{X}}_{k|k-1,1} & =F_{k,1}\cdot \Delta {\hat{X}}_{k-1,1}\\ P_{k|k-1,1} & =F_{k,1}\cdot P_{k-1,1}\cdot F_{k,1}+Q_{k-1,1} \end{array},$$
  where $\Delta {\hat{X}}_{k|k-1,1}$ is the priori state estimation, and $P_{k|k-1,1}$ is the priori estimation covariance matrix calculated by projecting the error covariance ahead.

- Correction

(30) $$K_{k,1}=P_{k|k-1,1}H_{k,1}^{T}{\left(H_{k,1}P_{k|k-1,1}H_{k,1}^{T}+R_{k,1}\right)}^{-1},$$

(31) $$\begin{array}{cc} \Delta {\hat{X}}_{k,1} & =\Delta {\hat{X}}_{k|k-1,1}+K_{k,1}\left(Z_{k,1}-H_{k,1}\Delta {\hat{X}}_{k|k-1,1}\right)\\ P_{k,1} & =\left(I_{4\times 4}-K_{k,1}H_{k,1}\right)P_{k|k-1,1} \end{array}.$$

In Equations (30) and (31), $K_{k,1}$ denotes KF gains, which is the weight of the difference between the received and predicted measurements. The KF gains are then exploited to estimate the posterior state estimation $\Delta {\hat{X}}_{k,1}$ by including the measurement $Z_{k,1}$. The posteriori estimation of the covariance matrix can be derived from $P_{k|k-1,1}$. Finally, the posterior state estimations $\Delta {\hat{\tau }}_{k,1}$ and $\Delta {\hat{f}}_{k,1}$ of each channel are input into the navigation filter as measurements.

### 4.3. Navigation Filter Model

For PLPS, the posterior estimations of the pre-filter can only reflect the error before the end of the current active timeslot. Thus, there is likely to be a time difference among the measurements sampling time of the navigation filter, as shown in the gray shade in Figure 4. Moreover, since the active timeslot of each pseudolite is pseudo-random and does not overlap, the time difference is time-varying. If the posterior estimations of the pre-filter are directly utilized as the measurements of the navigation filter, the measurement errors will increase, reducing the tracking performance of VTL. A predicted measurements module is added into the navigation filter in this paper to solve this problem, as shown in Figure 5. The mathematical model of the predicted measurements module is described as follows.

(32) $$\begin{array}{cc} \Delta \rho^{i} & =\lambda_{c}\cdot \left(\Delta {\hat{\tau }}_{k}^{i}+\beta \Delta {\hat{f}}_{k}^{i}\Delta t_{k}^{i}+\frac{1}{2}\beta \Delta {\hat{a}}_{k}^{i}\Delta t_{k}^{i}\right)\\ \Delta {\dot{\rho }}^{i} & =\lambda_{r}\cdot \left(\Delta {\hat{f}}_{k}^{i}+\Delta {\hat{a}}_{k}^{i}\Delta t_{k}^{i}\right) \end{array},$$

where the superscript *i* indicates the *i*-th channel. $\lambda_{c}$ and $\lambda_{r}$ are the wavelength of pseudorandom-code and radio frequency carrier, respectively. $\Delta \rho^{i}$ and $\Delta {\dot{\rho }}^{i}$ represent pseudo-range and pseudo-range rate errors that are the measurements of the navigation filter. $\Delta t_{k}^{i}$ denotes the intervals between the update time of the pre-filter of each channel and the input sampling time of the navigation filter.

The measurement vector of the navigation filter is

(33) $$Z_{k,2}={\left[\Delta \rho^{1},\Delta \rho^{2},\cdots ,\Delta \rho^{N},\Delta {\dot{\rho }}^{1},\Delta {\dot{\rho }}^{2},\cdots ,\Delta {\dot{\rho }}^{N}\right]}_{k}^{T},$$

where subscript 2 and *k* indicate the navigation filter and the *k*-th epoch.

The system state vector consists of eight errors and is described as

(34) $$\Delta X_{k,2}={\left[\Delta x,\Delta y,\Delta z,\Delta v_{x},\Delta v_{y},\Delta v_{z},\Delta b,\Delta d\right]}_{k,2}^{T},$$

where Δx, Δy, Δz, and $\Delta v_{x}$, $\Delta v_{y}$, $\Delta v_{z}$ are the receiver position and velocity errors, Δb and Δd are clock bias and drift errors. The system state can be described as a first-order Gauss-Markov process, which is given by

(35) $$\Delta X_{k,2}=F_{k,2}\cdot \Delta X_{k-1,2}+w_{k,2},$$

where $w_{k,2}$ is process noise, and its covariance matrix is $Q_{k-1,2}$. $F_{k,2}$ is the transition matrix, which can be expressed as follows.

(36) $$F_{k-1,2}={\left[\begin{array}{ccc} I_{3\times 3} & T_{f}\cdot I_{3\times 3} & 0_{3\times 2}\\ 0_{3\times 3} & I_{3\times 3} & 0_{3\times 2}\\ 0_{2\times 3} & 0_{2\times 3} & K \end{array}\right]}_{8\times 8},$$

where $T_{f}$ is the frame period of PRPS defined in Equation (3), $I_{3\times 3}$ represents the third order identity matrix, and K is

(37) $$K=\left[\begin{array}{cc} 1 & T_{f}\\ 0 & 1 \end{array}\right].$$

The pseudorange equation can be modeled as follows for the *i*-th pseudolite.

(38) $$\rho_{k}^{i}=r_{k}^{i}+b_{k}+n_{k}^{i},$$

where $\rho_{k}^{i}$ is the corrected pseudorange. *b* represents receiver clock bias. $n_{k}^{i}$ represents pseudo-range noise. $r_{k}^{i}$ indicates the distance between the receiver and pseudolite, which is given by

(39) $$r_{k}^{i}=\sqrt{{\left(x^{i}-x_{k}\right)}^{2}+{\left(y^{i}-y_{k}\right)}^{2}+{\left(z^{i}-z_{k}\right)}^{2}},$$

where $x^{i}$, $y^{i}$, and $z^{i}$ represent the position of *i*-th pseudolite. $x_{k}$, $y_{k}$, $z_{k}$ indicates the receiver position at *k*-th epoch. Because pseudolites are built on the ground, this paper assumes that their positions are fixed. Therefore, the navigation filter can omit the process of estimating the pseudolite position during every epoch.

At epoch *k*, a first-order Taylor’s expansion is used to linearize the relationship between the state and measurement vectors. According to Equations (38) and (39), $\Delta \rho_{k}^{i}$ is

(40) $$\Delta \rho_{k}^{i}=-l_{x,k}^{i}\cdot \Delta x_{k}-l_{y,k}^{i}\cdot \Delta y_{k}-l_{z,k}^{i}\cdot \Delta z_{k}+\Delta b_{k},$$

where $l_{k}^{i}={\left[l_{x}^{i},\;\;l_{y}^{i},\;\;l_{z}^{i}\right]}_{k}^{T}$ is the LOS projection from the receiver to *i*-th pseudolite and can be expressed as:

(41) $$\left\{\begin{array}{c} l_{x,k}^{i}=\left(x^{i}-x_{k}\right)/r_{k}^{i}\\ l_{y,k}^{i}=\left(y^{i}-y_{k}\right)/r_{k}^{i}\\ l_{z,k}^{i}=\left(z^{i}-z_{k}\right)/r_{k}^{i} \end{array}.\right.$$

Similarly, the pseudorange-rate error $\Delta {\dot{\rho }}_{k}^{i}$ is given by

(42) $$\Delta {\dot{\rho }}_{k}^{i}=-l_{k}^{i}\cdot \Delta v_{k}+\Delta d_{k},$$

where $v_{k}={\left[\Delta v_{x},\Delta v_{y},\Delta v_{z}\right]}_{k}^{T}$.

According to Equations (40) and (42), the measurement equation can be obtained as:

(43) $$Z_{k,2}=H_{k,2}\cdot \Delta X_{k,2}+v_{k,2},$$

where $v_{k,2}$ is the measurement noise, and its covariance matrix is $R_{k,2}$. The measurement matrix $H_{k,2}$ is given by

(44) $$H_{k,2}={\left[\begin{array}{cccccccc} -l_{x}^{1} & -l_{y}^{1} & -l_{z}^{1} & 0 & 0 & 0 & 1 & 0\\ -l_{x}^{2} & -l_{y}^{2} & -l_{z}^{2} & 0 & 0 & 0 & 1 & 0\\ \vdots & \vdots & \vdots & \vdots & \vdots & \vdots & \vdots & \vdots \\ -l_{x}^{N} & -l_{y}^{N} & -l_{z}^{N} & 0 & 0 & 0 & 1 & 0\\ 0 & 0 & 0 & -l_{x}^{1} & -l_{y}^{1} & -l_{z}^{1} & 0 & 1\\ 0 & 0 & 0 & -l_{x}^{2} & -l_{y}^{2} & -l_{z}^{2} & 0 & 1\\ \vdots & \vdots & \vdots & \vdots & \vdots & \vdots & \vdots & \vdots \\ 0 & 0 & 0 & -l_{x}^{N} & -l_{y}^{N} & -l_{z}^{N} & 0 & 1 \end{array}\right]}_{2N\times 8}.$$

Then, the posterior pseudorange and pseudorange-rate error estimations $\Delta {\hat{Y}}_{k}$ can be achived by:

(45) $$\Delta Y_{k}=H_{k,2}\cdot \Delta X_{k,2}.$$

The feedback corrections to the NCO of each channel is calculated as follows:

(46) $$\begin{array}{cc} \Delta \tau_{\mathrm{NCO},k}^{i} & =\left({\hat{\rho }}_{k}^{i}-{\tilde{\rho }}_{k}^{i}\right)/\lambda_{c}\\ \Delta f_{\mathrm{NCO},k}^{i} & =\left({\hat{\dot{\rho }}}_{k}^{i}-{\tilde{\dot{\rho }}}_{k}^{i}\right)/\lambda_{r} \end{array},$$

where ${\hat{\rho }}_{k}^{i}$ and ${\tilde{\rho }}_{k}^{i}$ are the estimated and measured pseudo-range at *k*-th epoch. ${\hat{\dot{\rho }}}_{k}^{i}$ and ${\tilde{\dot{\rho }}}_{k}^{i}$ are the estimated and measured pseudo-range rate at *k*-th epoch.

The process of the PM navigation filtering can be summarized as follows, based on the system dynamic and measurement models established above.

- Step1: Predict the measurements according to Equation (32) so that they can be sampled at the same time.
- Step2: Estimate the priori state: $\Delta {\hat{X}}_{k|k-1,2}=F_{k,2}\cdot \Delta {\hat{X}}_{k-1,2}$.
- Step3: Calculate the priori estimation covariance: $P_{k|k-1,2}=F_{k,2}\cdot P_{k-1,2}\cdot F_{k,2}+Q_{k-1,2}$.
- Step4: Calculate the Kalman gain: $K_{k,2}=P_{k|k-1,2}H_{k,2}^{T}{\left(H_{k,2}P_{k|k-1,2}H_{k,2}^{T}+R_{k,2}\right)}^{-1}$.
- Step5: Estimate the posterior state: $\Delta {\hat{X}}_{k,2}=\Delta {\hat{X}}_{k|k-1,2}+K_{k,2}\left(Z_{k,2}-H_{k,2}\Delta {\hat{X}}_{k|k-1,2}\right)$.
- Step6: Compute the posterior estimation covariance: $P_{k,2}=\left(I_{8\times 8}-K_{k,2}H_{k,2}\right)P_{k|k-1,2}$.
- Step7: LOS Projection: $\Delta {\hat{Y}}_{k}=H_{k,2}\cdot \Delta {\hat{X}}_{k,2}$.
- Step8: Feedback to NCO: as described in Equation (46).

## 5. Simulation and Results

In this section, a simulation is carried out to compare the performance of the proposed IUPPM-VTL with traditional VTL and IUP-VTL.

### 5.1. Simulation Setup

**Figure 6:**
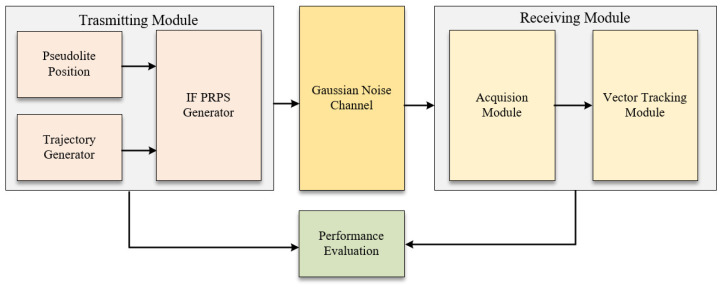
Block diagram of the simulation scheme.

Figure 6 shows the block diagram of the self-developed simulation scheme. First, the position and velocity of the receiver are preset in the trajectory generator. Then, the IF PRPS can be produced according to the fixed pseudolite position. This paper assumes that there is no multipath signal and that the pseudolite net is synchronous. Therefore, the PRPS is transmitted through the Gaussian channel, and it will be captured and tracked by the receiving module. Finally, the position, velocity, doppler, and code phase error results are used to evaluate the performance of different vector tracking structures.

The simulation in this paper is established on a self-defined XYZ coordinate system. This process does not affect the evaluation of vector tracking performance but avoids the transformation between coordinate systems. The parameters of the PRPS are listed in Table 1. The total number of pseudolite is 10 in the simulation.

**Figure 7:**
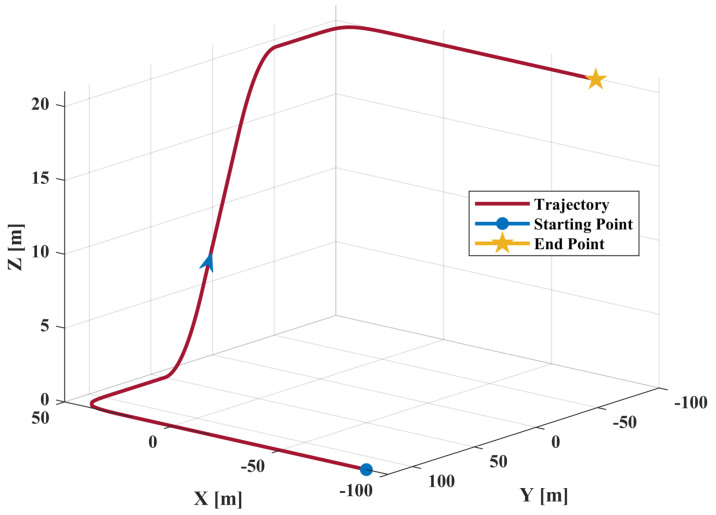
Reference trajectory.

**Figure 8:**
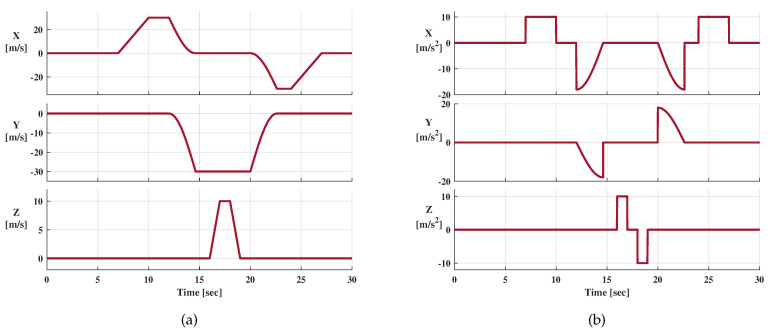
Reference dynamic. (**a**) Reference velocity. (**b**) Reference acceleration.

The reference trajectory is shown in Figure 7, the blue dot is the starting point, the blue arrow represents the direction of motion, and the yellow five-pointed star is the endpoint. The reference velocity and acceleration are presented in Figure 8a,b, respectively.

The dynamic process of the receiver is described as follows:

1. The receiver is in a static state during 0∼7 s, and the relative coordinate of the starting point is [−90,120,0] (m).
2. Conduct a constant acceleration movement in the +x direction with an acceleration of 10 m/s and a duration of 2 s.
3. Stop acceleration and maintain a constant velocity state for 1 s.
4. Make a 1/4 circular motion with a circle radius of 50 m.
5. The receiver moves toward the −y direction and keeps moving at a constant speed for 1.382 s.
6. Apply a constant acceleration in the +z direction. The acceleration is 10 m/s, and the duration is 1 s.
7. Stop acceleration and maintain a constant velocity state for 1 s.
8. Perform a deceleration movement in the +z direction until $V_{z}=0$ and the acceleration is −10 m/s.
9. Keep a uniform speed in the −y direction for 1 s.
10. Make a 1/4 circular motion with a circle radius of 50 m.
11. The receiver moves toward the −x direction and keeps moving at a constant speed for 1.382 s.
12. Perform a deceleration movement in the −x direction until $V_{x}=0$ and the acceleration is −10 m/s.
13. The receiver remains stationary for 3 s.

The dynamic process described above lasts for a total time of 30 s and the coordinate of end point is [−71.5,−98.3,20.0] (m).

### 5.2. Simulation Results

This paper pays more attention to the influence of the PLPS on tracking and position accuracy, weakening the impact of factors, such as the received signal power fluctuation, with the distance variation. Thus, the received signal power is set as fixed. The carrier-to-noise ratio ($C/N_{0}$) of IF PRPS is set to 50 dB · Hz in this simulation scenario. Next, three different VTL structures are applied to track the preset IF PRPS. Finally, the tracking and navigation results are analyzed and compared.

**Figure 9:**
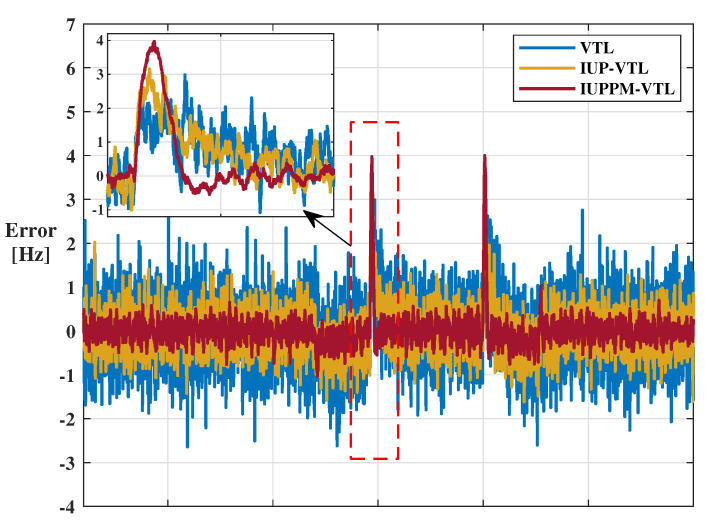
Doppler error of the 10th-Pseudilite.

**Figure 10:**
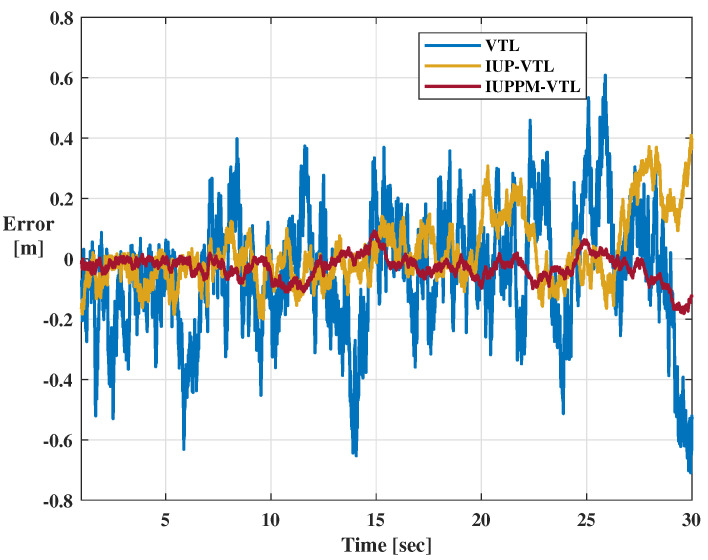
Code phase error of the 10th-Pseudilite.

**Figure 11:**
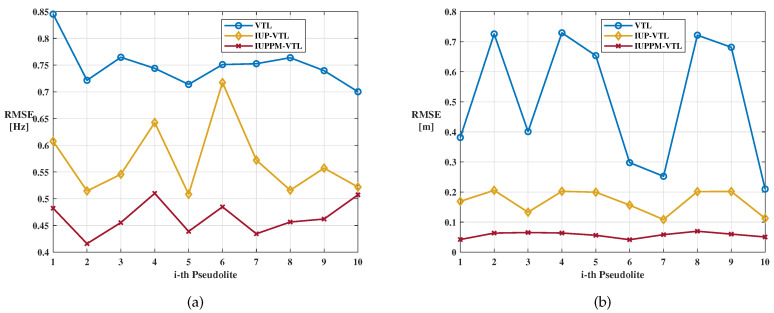
Tracking results of all pseudolites. (**a**) Doppler error of all pseudolites. (**b**) Code phase error of all pseudolites.

The tracking errors of VTL mainly refer to doppler error and code phase error, which are obtained by the difference between the local replica signal and the received signal. Figure 9 and Figure 10 shows the tracking errors of the 10th-Pseudilite. The blue, yellow, and red lines represent the position errors of VTL, IUP-VTL, and IUPPM-VTL algorithms, respectively. The dashed box shown in Figure 9 describes the peak caused by sudden changes in acceleration. From the enlarged view, it can be seen that the IUPPM-VTL can respond to acceleration changes faster. The root mean square errors (RMSE) of doppler and code phase are calculated and recorded in Table 2. It is illustrated that the IUPPM-VTL has the best tracking accuracy throughout the tracking process. The tracking results of all pseudolites are demonstrated in Figure 11a,b.

**Figure 12:**
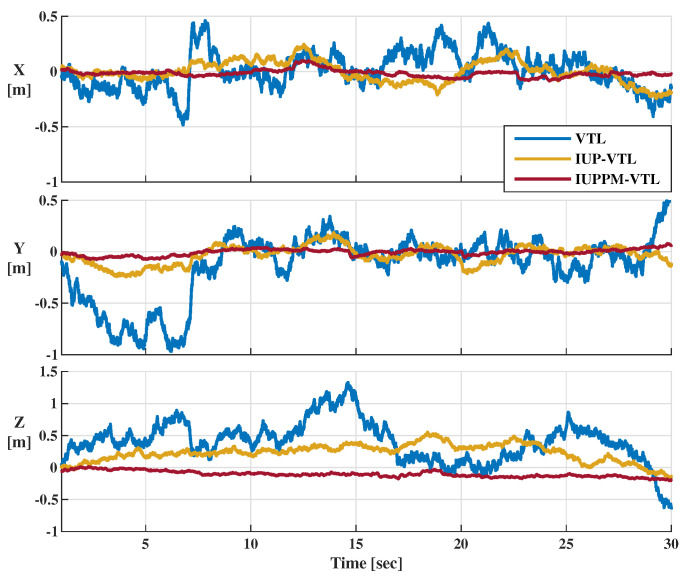
Position errors of three VTL structures in XYZ directions.

The position error is the difference between the position result and the reference trajectory shown in Figure 7. Figure 12 shows the position errors in XYZ directions. Because the IUPPM-VTL adopts a more accurate model, it performs a slighter jitter in position errors than VTL and IUP-VTL. The RMSEs of the position errors of VTL, IUP-VTL, and IUPPM-VTL are 0.63 m, 0.30 m, and 0.12 m, respectively. Hence, the proposed IUPPM-VTL performs best in position precision.

**Figure 13:**
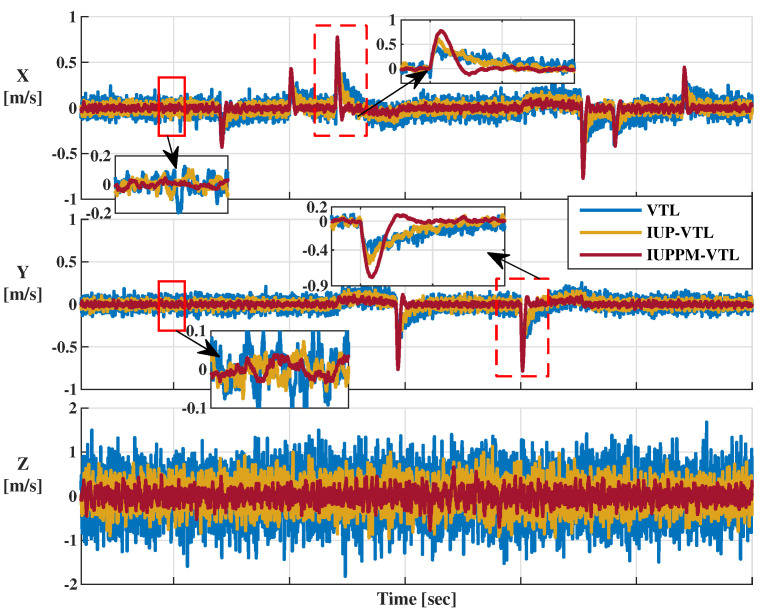
Velocity errors of three VTL structures in XYZ directions.

The velocity error is the difference between the velocity result and the reference velocity shown in Figure 8. Figure 13 shows the velocity errors in XYZ directions. The enlarged views of the solid line boxes demonstrate the velocity errors at the steady-state. The velocity errors at the abrupt acceleration in the X and Y directions are shown in the dotted box (details are shown in the enlarged version). When adopting the IUPPM-VTL, the sudden change in acceleration has a shorter disturbance time to the speed estimation than VTL and IUP-VTL because the proposed IUPPM-VTL has higher doppler tracking accuracy and more robust acceleration response capability. The RMSEs of the velocity errors of VTL, IUP-VTL, and IUPPM-VTL are 0.50 m/s, 0.40 m/s, and 0.24 m/s, respectively. Thus, the proposed IUPPM-VTL significantly improves velocity accuracy.

## 6. Conclusions

PLPS generally adopts a pseudo-random pulsing signal structure to mitigate the near-far effect. This signal pattern leads to irregular pulsing periods in the tracking loop when estimating the code phase, doppler, and carrier phase. However, traditional VTL is established on a continuous CDMA architecture. Thus, there exist two problems when directly adopting VTL to tracking PRPS.

- The discriminator model is affected by irregular pulsing periods in PLPS.
- The sampling time of the navigation filter inputs is inconsistent and time-varying in PLPS.

They will cause jitter degradation and biases in tracking PRPS. Thus, traditional VTL is incompatible with tracking PRPS.

Therefore, this paper proposes an optimized VTL architecture that includes IUP pre-filters and a navigation filter based on PM. According to the timeslot intervals, the IUP pre-filter based KF adjusts the update cycles, avoiding the impact of the pseudo-random pulsing signal. So, it can estimate the measurement accurately. Meanwhile, the navigation filter predicts the measurements of each pre-filter to the same sampling time. So, the measurements can reflect the errors of the sampling time, reducing the measurement noise of the navigation filter.

Additionally, a simulation test is carried out to compare the proposed IUPPM-VTL with traditional VTL based pre-filter and IUP-VTL based IUP-prefilter. The reference trajectory includes static, uniform acceleration, uniform speed, turning, and uniform deceleration. The maximum speed is 31.6 m/s, and the maximum acceleration is 18 m/s^2^. Therefore, this comprehensive scenario can better evaluate the performance of the VTLs. The results show that the proposed IUPPM-VTL has a higher tracking accuracy than IUP-VTL and VTL. Meanwhile, their position errors are 0.12 m, 0.30 m, and 0.63 m, and their velocity errors are 0.24 m/s, 0.40 m/s, and 0.50 m/s. Thus, the proposed IUPPM-VTL performs the highest position and velocity precision among the three VTLs. In future work, it is necessary to concentrate on implementing and applying this method to real-time PRPS.

## Figures and Tables

**Table 1:** Main parameters of PRPS.

RF ($f_{c}$)	1575.42 MHz
IF ($f_{IF}$)	24 MHz
code frequency	10.23 MHz
code length	1023 chips
duty cycle of the pulse pattern (*d*)	0.1
Timeslot Period ($T_{s}$)	0.1 ms
Frame Period ($T_{f}$)	1 ms
Number of Timeslots in one Frame ($N_{s}$)	10
Number of Frames ($N_{f}$)	200
Super Frame Period ($T_{p}$)	200 ms

**Table 2:** Comparision of doppler and code phase errors of the 10th-Pseudilite.

RMSE	VTL	IUP-VTL	IUPPM-VTL
Doppler [Hz]	0.7001	0.5218	0.5073
Code Phase [m]	0.2097	0.1124	0.0505
